# Aryl Hydrocarbon Receptor Deficiency Impairs Intestinal Barrier Development, While 2′-Fucosyllactose Reduces Paracellular Permeability in Caco-2 Cells

**DOI:** 10.3390/toxins18100451

**Published:** 2026-10-15

**Authors:** Avra Melina Madella, Carlon Catharina Emma van Gerven, Elena Sendino Garví, Jeroen van Bergenhenegouwen, Johan Garssen, Rosalinde Masereeuw, Saskia Adriana Overbeek

**Affiliations:** 1 Division of Pharmacology, Utrecht Institute for Pharmaceutical Sciences, Utrecht University, 3584 CG Utrecht, The Netherlandsj.garssen@uu.nl (J.G.); r.masereeuw@uu.nl (R.M.); 2 Danone Nutricia Research, 3584 CT Utrecht, The Netherlands

**Keywords:** 2′-FL, AhR, barrier dysfunction, human milk oligosaccharides, intestinal homeostasis, uremia

## Abstract

Chronic kidney disease (CKD) is increasingly linked to intestinal barrier dysfunction, which contributes to systemic inflammation and disease progression. We investigated whether 2′-fucosyllactose (2′-FL), a human milk oligosaccharide, could modulate intestinal epithelial barrier function under uremic toxin exposure and examined the role of the aryl hydrocarbon receptor (AhR). Using wild-type (WT) and AhR knockout (AhR KO) Caco-2 cell lines, we assessed barrier function and cellular responses through transepithelial electrical resistance (TEER), paracellular permeability (Papp), tight junction (TJ) gene expression, AhR target gene activation, and metabolic assays. AhR deficiency impaired epithelial maturation, confirming its role in barrier development. Short-term exposure to uremic toxins (UTs) did not significantly affect TEER or paracellular permeability under the conditions tested. However, 2′-FL reduced paracellular permeability in both WT and AhR KO cells in the presence of FCS. These effects occurred without changes in TJ gene expression, indicating mechanisms beyond transcriptional regulation. AhR target genes were not induced by UTs, suggesting limited receptor activation, and metabolic activity was unaffected. Overall, 2′-FL reduced paracellular permeability under selected experimental conditions, while the impaired barrier development observed in AhR-deficient cells supports a role for AhR in intestinal epithelial barrier development.

## 1. Introduction

Chronic kidney disease (CKD) is a progressive condition that affects over 10% of the global population, translating to more than 800 million people worldwide [1]. While existing treatments can slow or sometimes halt the progression of CKD, most patients continue to experience worsening kidney function. Additionally, treatments are often not fully accessible or optimally utilized across diverse populations.

The incidence of CKD is determined by both non-modifiable and modifiable risk factors [2,3]. Non-modifiable factors include genetic variants, low birth weight, and aging, whereas modifiable factors encompass smoking, obesity, hypertension, diabetes, excessive alcohol consumption, heavy metal exposure, and overuse of analgesics [3]. Regardless of type, these factors can promote kidney damage through injury to diverse cell populations in the glomerulus (e.g., podocytes, mesangial cells, endothelial cells) and the tubulointerstitium (e.g., tubular cells, endothelial cells, fibroblasts), thereby contributing to disease onset and progression.

Emerging evidence suggests that CKD pathology extends beyond the kidneys themselves [4]. Patients with CKD often exhibit intestinal dysbiosis, which refers to an imbalance in gut microbiota composition and function that favors proteolytic bacteria and the production of uremic metabolites [5]. This dysbiosis is commonly triggered by diet changes and altered host conditions due to disease and medications, leading to loss of mucosal homeostasis often accompanied by increased intestinal permeability or a “leaky gut [6,7]. Evidence supporting this includes the detection of orally ingested polyethylene glycol in the urine of patients with CKD, as well as the presence of bacterial DNA and endotoxins in their bloodstream, indicating systemic translocation of gut-derived microbes as a consequence of lost barrier integrity [8,9].

In CKD, increased intestinal permeability contributes to systemic inflammation and oxidative stress, exacerbating kidney injury and multi-organ damage [10]. The relationship between the gut and kidneys is bidirectional: while intestinal dysbiosis and increased permeability may accelerate CKD progression, the decline in kidney function and accumulation of uremic metabolites may lead to alterations in the gut microbiome. Although the exact mechanisms remain unclear, the growing body of evidence strongly supports the existence of a gut–kidney axis.

A critical mediator in this axis is the aryl hydrocarbon receptor (AhR), a nuclear receptor activated by both endogenous and exogenous ligands [11]. AhR is essential for kidney development and function as well as for maintaining intestinal homeostasis [12,13,14]. The heterogeneity in CKD progression indicated by differences in the rate of renal functional decline, the extent of fibrosis, inflammation, and oxidative stress, as well as the susceptibility of specific nephron segments, may be partly explained by variability in AhR activation and downstream signaling in response to different ligands. AhR engagement by certain agonists, including uremic toxins (UTs), such as indoxyl sulfate (IS) and indole-3-acetic acid (I3AA), non-steroidal anti-inflammatory drugs (NSAIDs), and specific tryptophan metabolites (such as kynurenine or kynurenic acid), has been shown to promote pro-inflammatory, pro-fibrotic, and oxidative pathways, thereby exacerbating kidney injury and accelerating disease progression [12]. In contrast, other ligands, such as quinoline derivatives and dietary molecules (e.g., resveratrol), can activate AhR-dependent cytoprotective and anti-inflammatory responses, suggesting a potential protective role in CKD [15].

While several therapeutic strategies targeting the colonic microenvironment in CKD have been explored, including modulating gut microbiota, blocking lipopolysaccharides, and reducing inflammation, few specifically address AhR signaling to support intestinal barrier function. This represents an important gap in our understanding of potential therapeutic targets for CKD management.

Given the emerging evidence supporting the gut–kidney axis and the role of AhR in CKD progression, this study explores whether interventions targeting the intestinal barrier can support epithelial barrier dysfunction during acute uremic toxin (UT) exposure. In particular, we investigated 2′-fucosyllactose (2′-FL), a human milk oligosaccharide that has been shown to enhance epithelial barrier integrity and maintain tight junction function, as well as to exert immunomodulatory effects in inflammatory models [16,17]. These dual properties make 2′-FL a promising candidate for counteracting CKD-associated gut barrier disruption, as it may both prevent the translocation of pro-inflammatory microbial products and modulate systemic inflammatory responses. By examining the potential of 2′-FL to support barrier function, this study aims to determine whether its effects are dependent on AhR signaling, providing insight into the mechanistic links between UT exposure, intestinal barrier dysfunction, and systemic inflammation in CKD.

## 2. Results

### 2.1. An AhR KO Line Was Successfully Created Using CRISPR-Cas9

CRISPR-Cas9 was used to generate a Caco-2 cell line with an AhR KO (Supplementary Figure S1a). DNA sequencing was conducted for individual clones to select a successful AhR KO and WT negative control. Sanger sequencing and Synthego analysis confirmed that CRISPR-Cas9 resulted in clear disruptions in the AhR gene at the cutting site of the guide RNA (gRNA; Supplementary Figure S1b,c). The effect of CRISPR-Cas9 on the eventual protein structures was predicted by TIDE analysis (https://tide.nki.nl Accessed 1 May 2024). Protein structure prediction could only be conducted for one allele of the AhR KO, as the second allele did not contain sufficient amino acid sequence for structural prediction.

The predicted protein sequence of the negative control remained intact, whereas the identified edits in the selected AhR KO clone were predicted to result in a truncated and structurally disrupted AhR protein (Supplementary Figure S1d,e). Therefore, these clones were selected as AhR WT and AhR KO human colonic cells for further experiments.

### 2.2. AhR Loss Impairs Intestinal Barrier Formation

**Figure 1:**
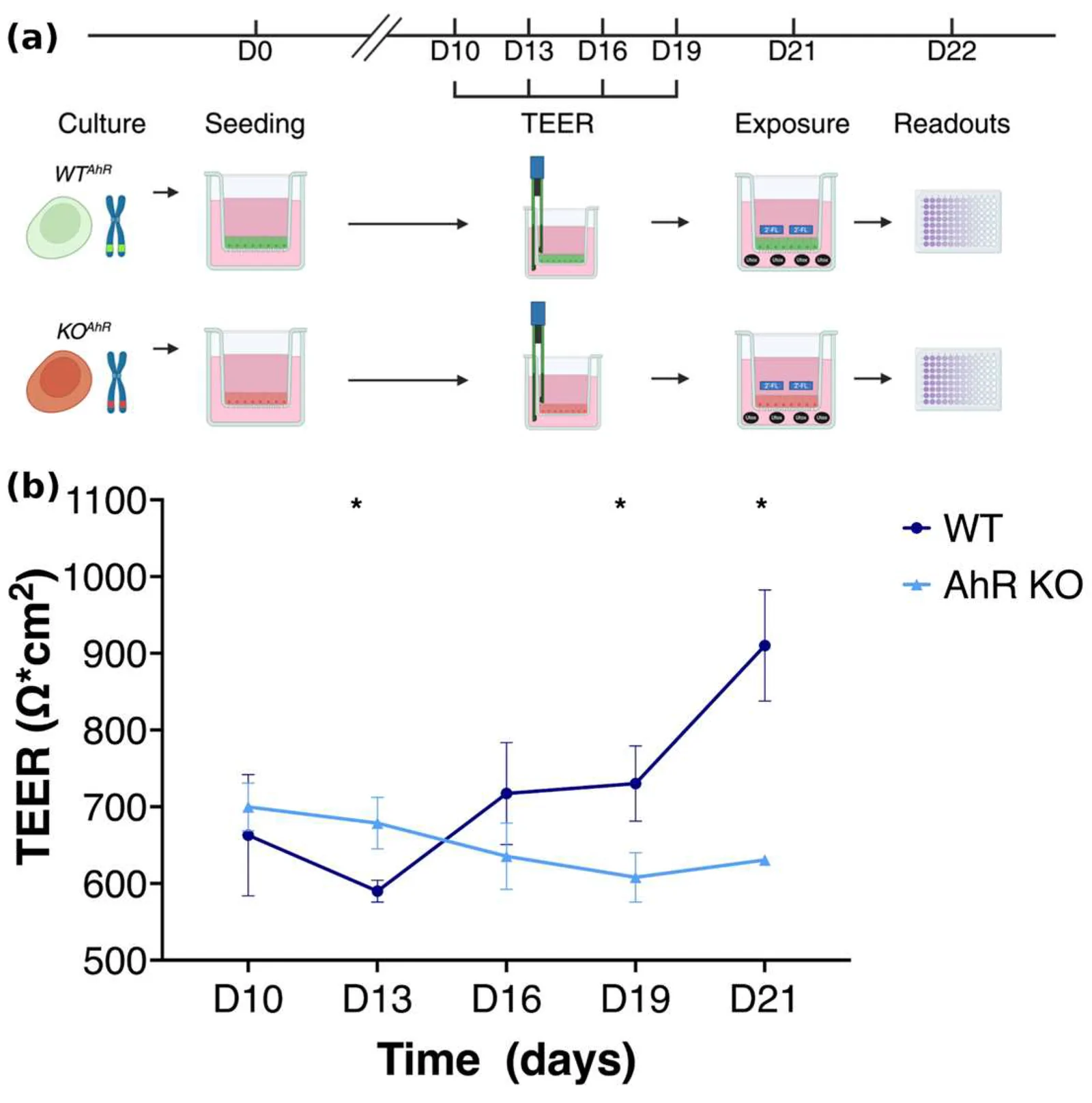
Impact of AhR deficiency on TEER in Caco-2 cells. (**a**) Schematic overview of the experimental setup used to assess TEER; (**b**) Time-course comparison of TEER development between AhR KO and WT Caco-2 cells. A mixed-effects analysis (REML) with Tukey’s post hoc analysis was conducted to establish the differences between the two cell types. Both cell type (*p* = 0.0006) and time duration (*p* = 0.0160) were found to have a significant impact. Significant differences in TEER values between the two cell lines were found on D13 (*p* = 0.0301), D19 (*p* = 0.0287), and D21 (*p* = 0.0207). Data are presented as mean ± SD. (*N* = 3, *n* = 2). * *p* < 0.05. Created in BioRender. Masereeuw, R. (2026) https://BioRender.com/qd10u95, accessed on 12 October 2026.

To assess the role of AhR in epithelial barrier development, TEER was monitored over a 21- day differentiation period in WT and AhR KO Caco-2 cells (Figure 1a). TEER values were significantly lower in AhR KO cells compared to WT cells, with significant differences on D13, D19, and D21 of differentiation (Figure 1b).

### 2.3. AhR KO and WT Cells Show Differential Responses Post 24 h Exposures to UTs and 2′-FL

**Figure 2:**
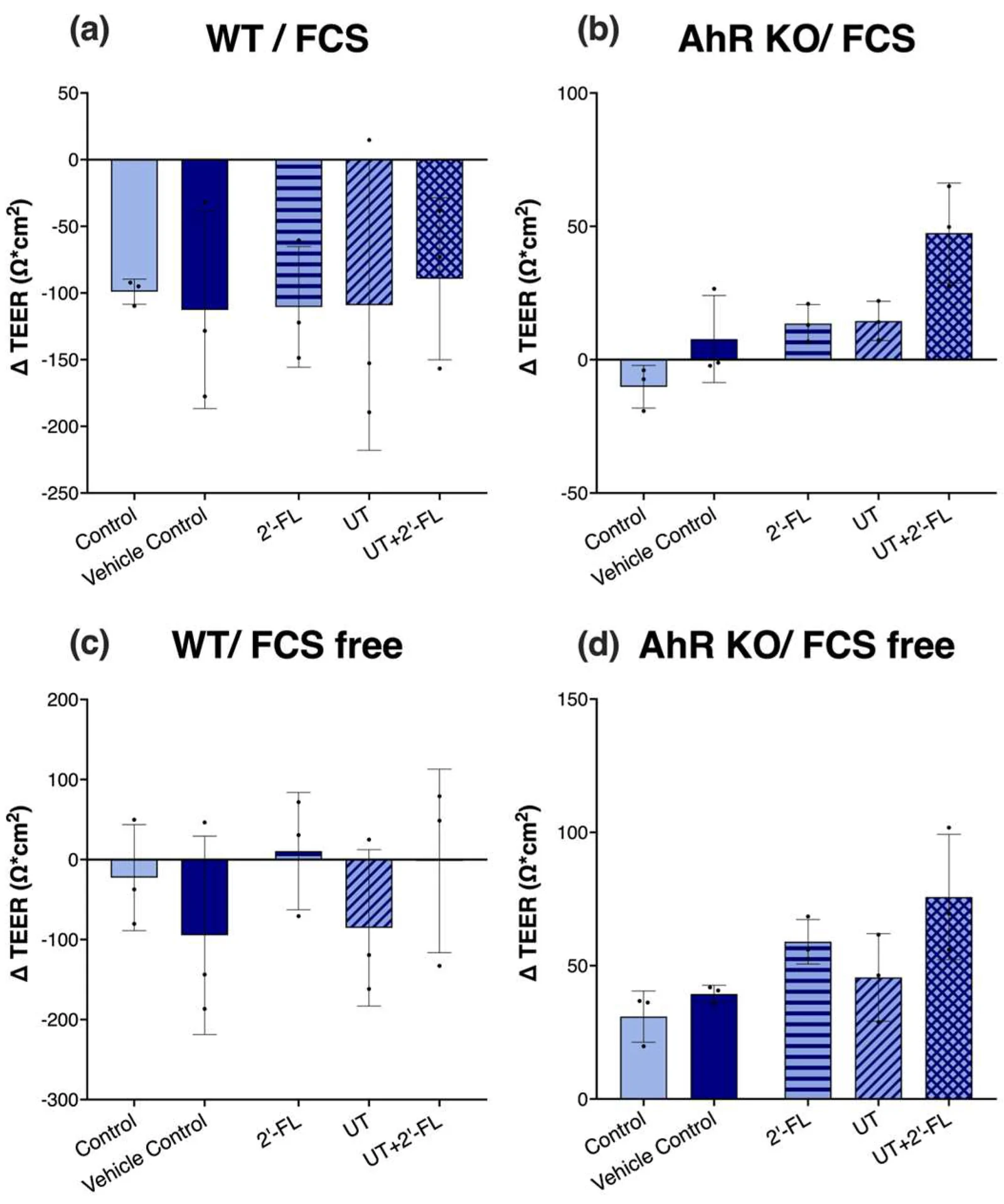
AhR knockout (KO) and wild-type (WT) Caco-2 cells show differential responses 24 h post-exposure. ΔTEER values are shown for WT (**a**,**c**) and AhR KO (**b**,**d**) cells under different exposures, in the presence (**a**,**b**) and absence (**c**,**d**) of FCS. Data are presented as mean ± SD (*N* = 3, *n* = 2). Within each cell type and FCS condition, treatment-specific effects were assessed using Friedman’s test with Dunn’s post hoc test. A significant overall effect was observed in AhR KO cells (*p* = 0.0151 for both presence and absence of FCS), and a trend was noted in WT cells in the absence of FCS (*p* = 0.0634). Post hoc analysis did not reveal significant differences between specific treatment groups.

Following 21-day differentiation, cells were exposed for 24 h to UTs, 2′-FL, or a combination under FCS and serum-free conditions (Figure 2). In WT cells, ΔTEER values were negative under FCS conditions (Figure 2a) and slightly positive under serum-free conditions following 2′-FL or combination treatment (Figure 2b) In AhR KO cells, ΔTEER values were predominantly positive under all conditions (Figure 2c,d).

### 2.4. 2′-FL Reduces Paracellular Permeability in Both WT and AhR KO Cells in the Presence of FCS

**Figure 3:**
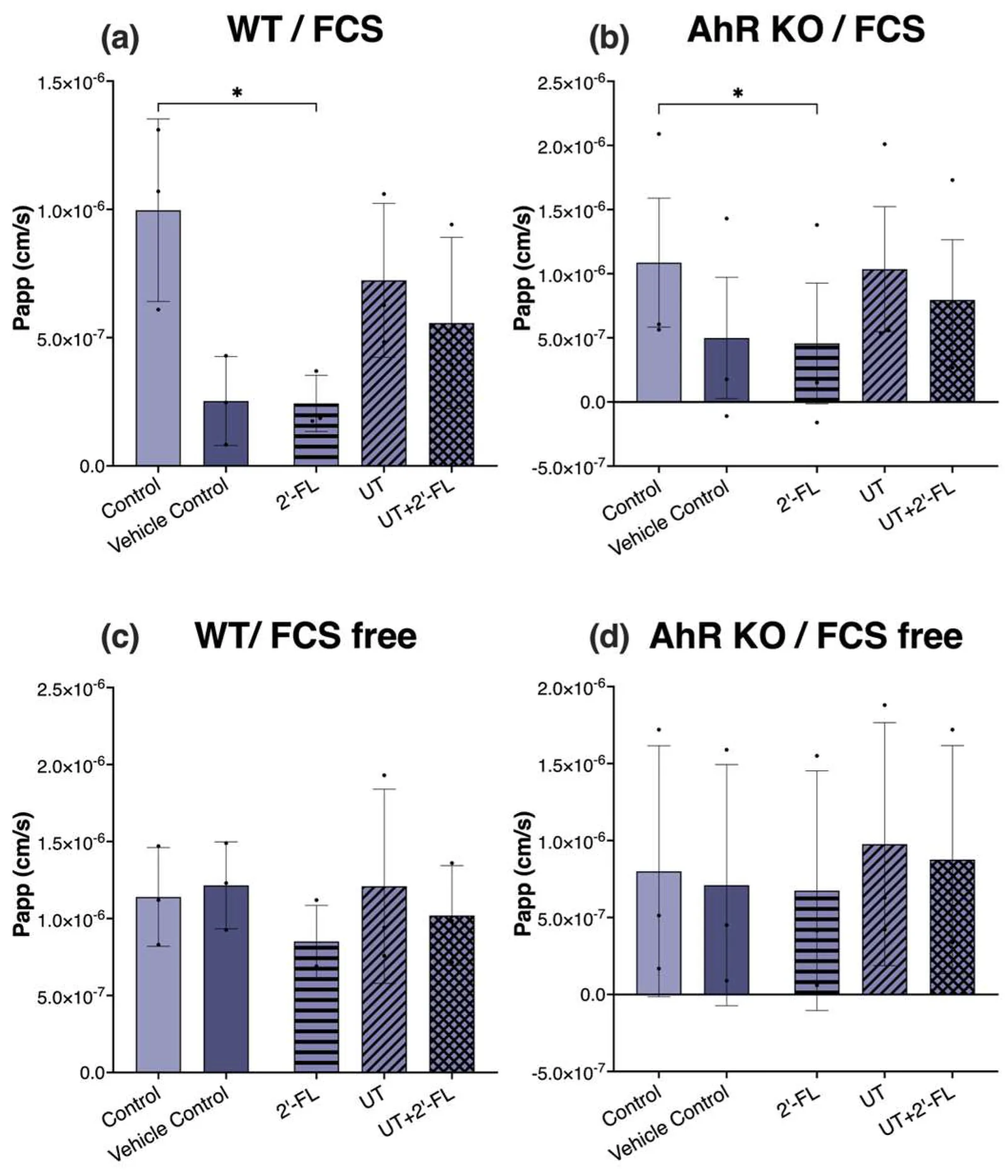
2′-FL reduces paracellular permeability in both WT and AhR KO Caco-2 cells in the presence of FCS. Apparent permeability is shown for WT (**a**,**c**) and AhR KO (**b**,**d**) cells under different exposures, in the presence (**a**,**b**) and absence (**c**,**d**) of FCS. Data are presented as mean ± SD (*N* = 3, *n* = 2). Within each cell type and FCS condition, treatment-specific effects were assessed using Friedman’s test with Dunn’s post hoc test. A significant overall effect was observed in WT cells in the presence of FCS (*p* = 0.0009), with post hoc analysis revealing a significant difference between 2′-FL and control (*p* = 0.0271). Similarly, AhR KO cells in the presence of FCS showed a significant overall effect (*p* < 0.0001) and a post hoc difference between 2′-FL and control (*p* = 0.0171). An overall effect was also observed in AhR KO cells in the absence of FCS (*p* = 0.0001), but post hoc analysis did not identify significant differences. * denotes *p* < 0.05.

Paracellular permeability was assessed using FITC-inulin. In WT cells with FCS, 2′-FL treatment showed lower permeability compared to control, while no clear differences were observed in the absence of FCS (Figure 3a,c). Similarly, in AhR KO cells, permeability was reduced with 2′-FL treatment in the presence of FCS, with no effect seen in the absence of FCS (Figure 3b,d).

### 2.5. Neither UTs nor 2′-FL Significantly Impact Tight-Junction Expression in WT or AhR KO Cells

**Figure 4:**
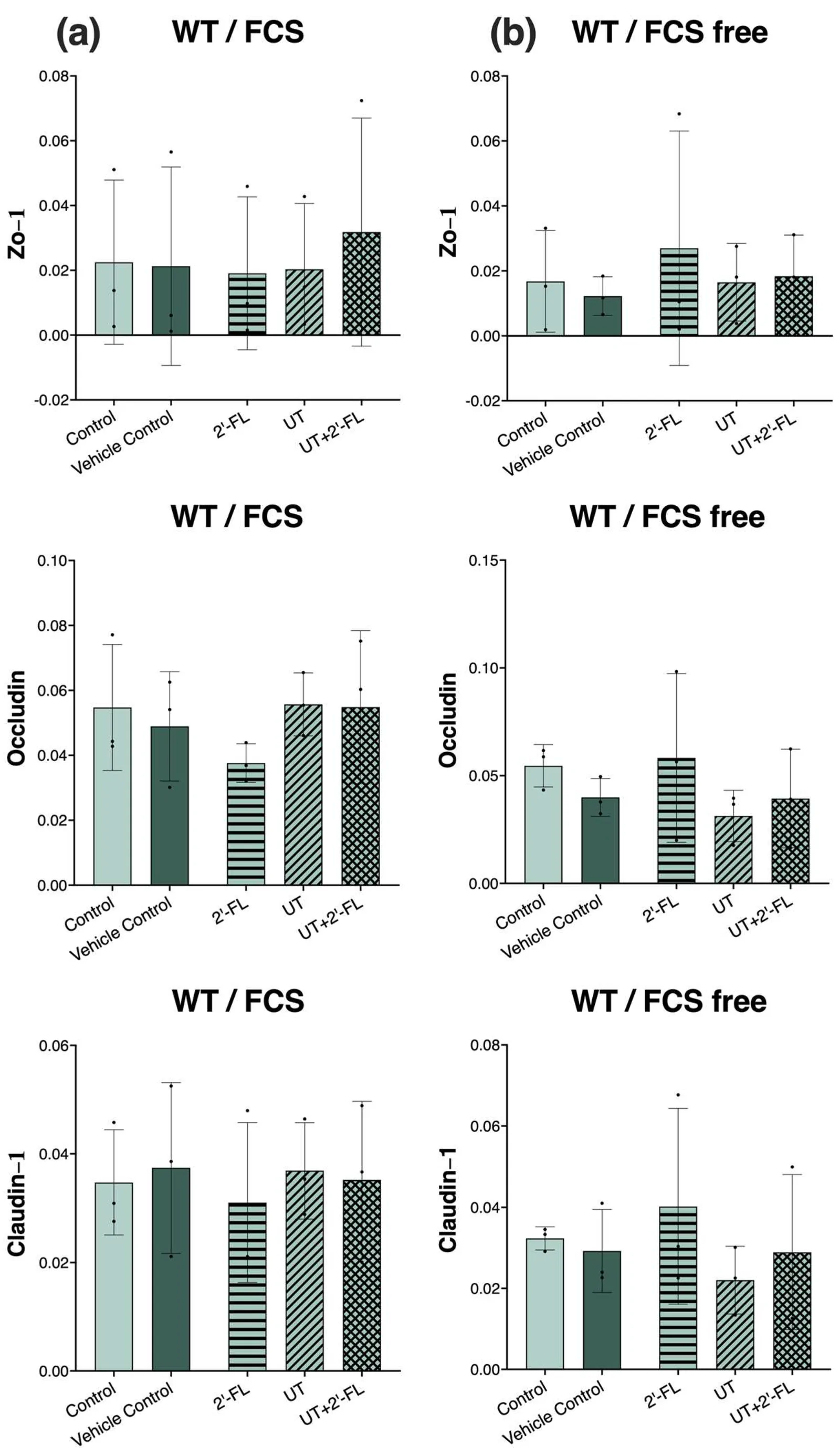
Neither UTs nor 2′-FL significantly impact tight-junction expression in WT Caco-2 cells. Relative tight junction expression is shown for WT cells under different exposures, in the presence (**a**) and absence (**b**) of FCS. mRNA gene expression was calculated using the 2^−ΔCT method and normalized to β-actin. Data are presented as mean ± SD (*N* = 3, *n* = 2). Within each cell type and FCS condition, treatment-specific effects were assessed using Friedman’s test with Dunn’s post hoc test. No significant effects were seen.

**Figure 5:**
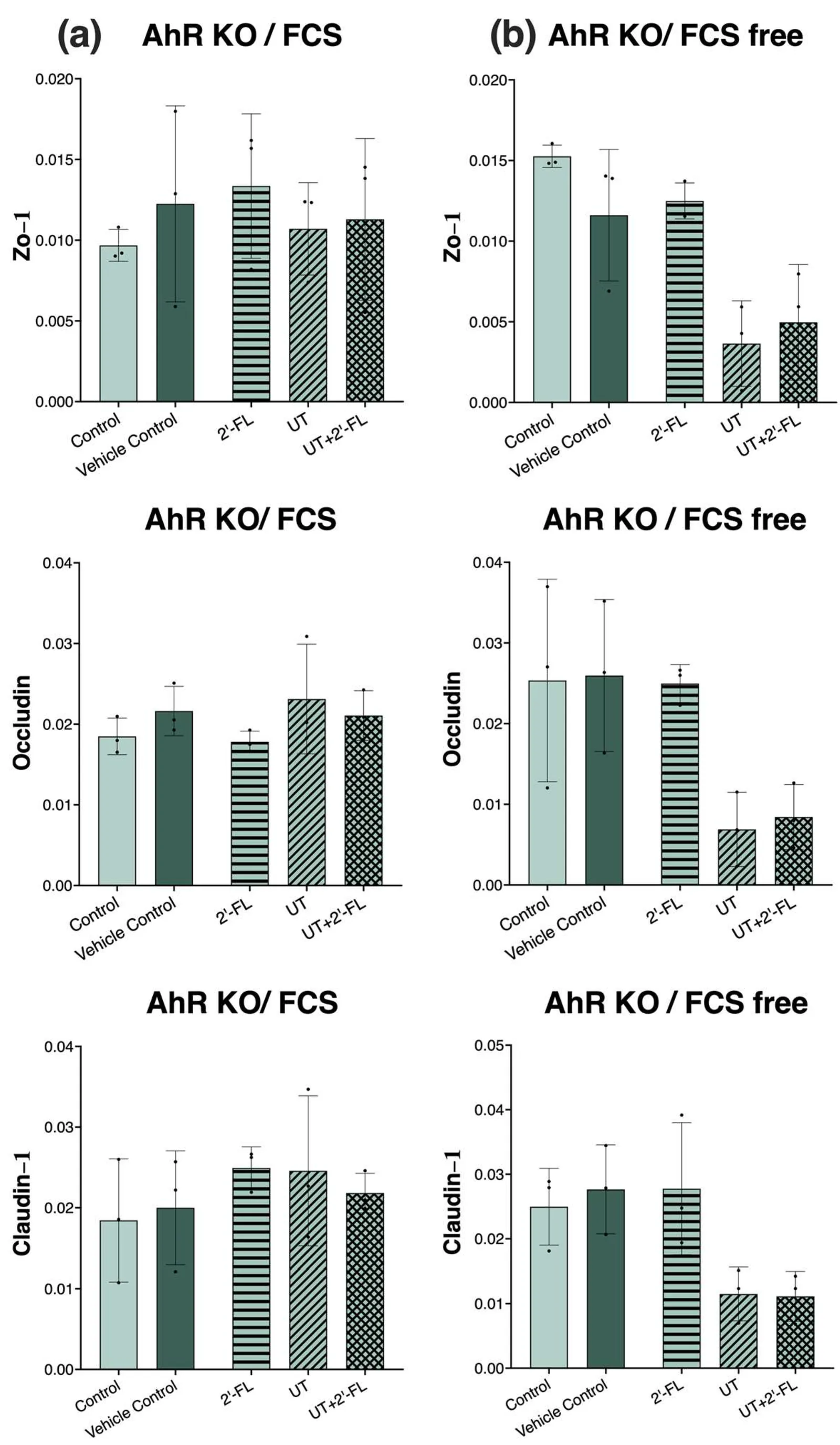
Neither UTs nor 2′-FL significantly impact tight-junction expression in AhR KO Caco-2 cells. Relative tight junction expression is shown for AhR KO cells under different exposures, in the presence (**a**) and absence (**b**) of FCS. mRNA gene expression was calculated using the 2^−ΔCT method and normalized to β-actin. Data are presented as mean ± SD (*N* = 3, *n* = 2). Within each cell type and FCS condition, treatment-specific effects were assessed using Friedman’s test with Dunn’s post hoc test. An overall significant effect was observed in AhR KO cells in the absence of FCS for ZO-1 (*p* = 0.0053), Claudin-1 (*p* = 0.0259), and a trend was seen for Occludin (*p* = 0.0559). In the presence of FCS, Occludin also showed a significant overall effect (*p* = 0.0259); however, post hoc analysis did not reveal significant differences between treatments, neither in the presence nor in the absence of FCS.

mRNA expression of ZO-1, Occludin, and Claudin-1 was measured to assess tight junction integrity. In both WT (Figure 4) and AhR KO cells (Figure 5), mRNA expression of all three genes was similar across treatments under both FCS and serum-free conditions.

### 2.6. UTs Do Not Activate AhR Target Genes CYP1A1 and HMOX-1

**Figure 6:**
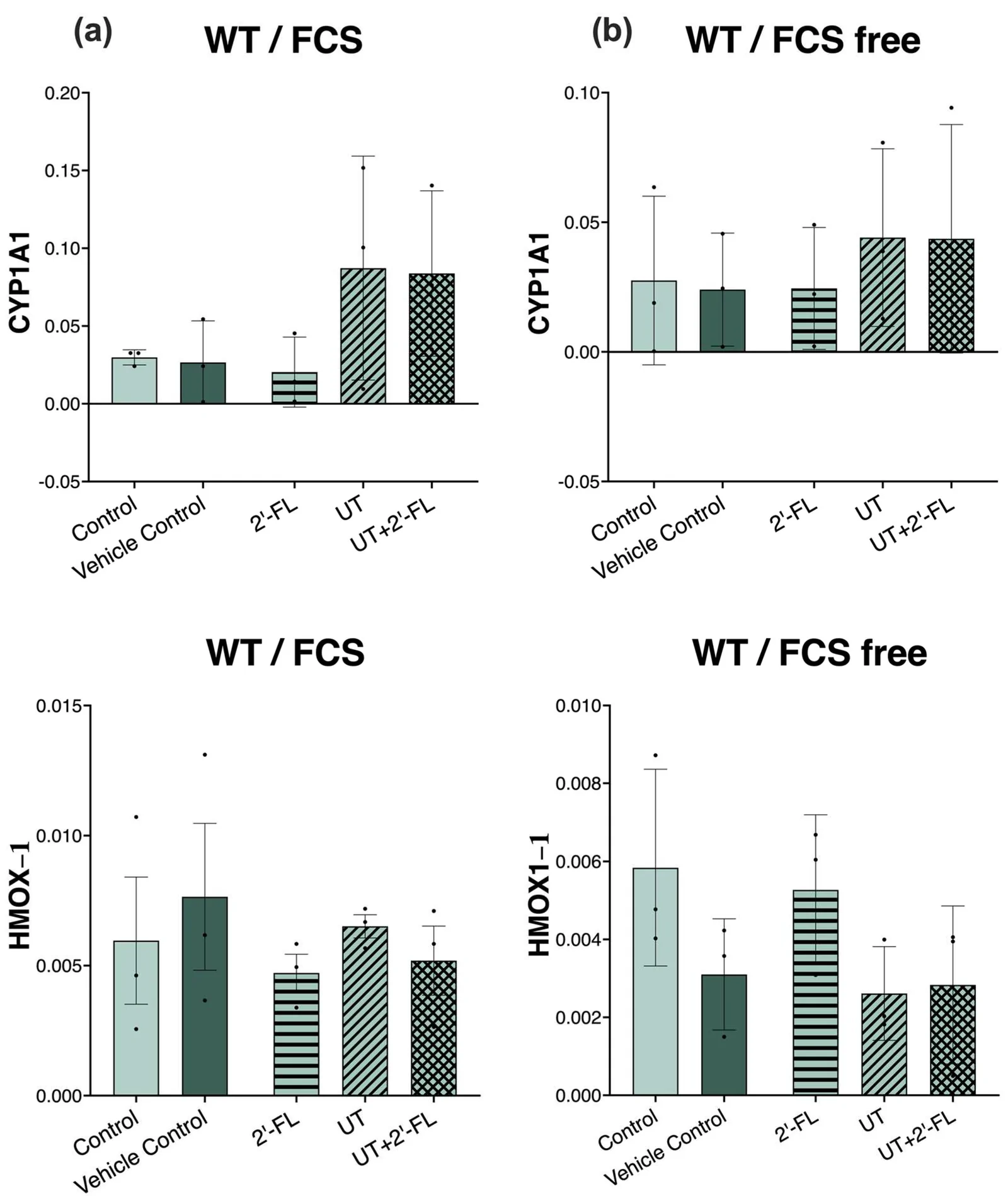
UTs do not activate AhR target genes CYP1A1 and HMOX-1 in WT Caco-2 cells. Relative mRNA expression of canonical AhR target genes is shown for WT Caco-2 cells under different exposures, in the presence (**a**) and absence (**b**) of FCS. mRNA expression was calculated using the 2^−ΔCT method and normalized to β-actin. Data are presented as mean ± SD (*N* = 3, *n* = 2). Within each cell type and FCS condition, treatment-specific effects were assessed using Friedman’s test with Dunn’s post hoc test.

**Figure 7:**
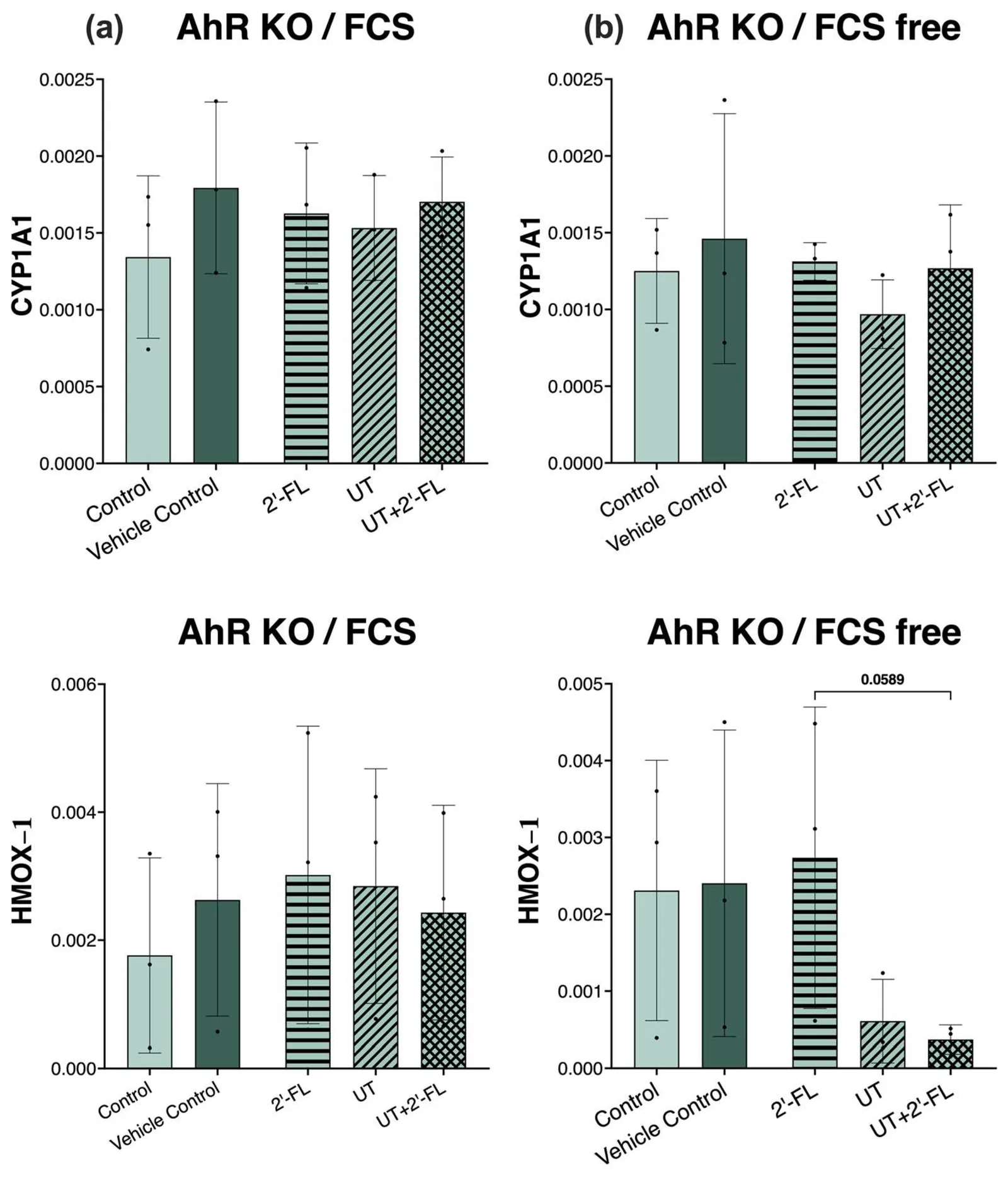
UTs do not activate AhR target genes CYP1A1 and HMOX-1 in AhR KO Caco-2 cells. Relative expression of canonical AhR target genes is shown for AhR KO Caco-2 cells under different exposures, in the presence (**a**) and absence (**b**) of FCS. Gene expression was calculated using the 2^−ΔCT method and normalized to β-actin. Data are presented as mean ± SD (*N* = 3, *n* = 2). Within each cell type and FCS condition, treatment-specific effects were assessed using Friedman’s test with Dunn’s post hoc test. An overall significant effect was observed in AhR KO cells both in the presence of FCS (*p* = 0.0376) and in the absence of FCS (*p* = 0.0078). Post hoc analysis revealed no significant differences between treatment groups in AhR KO cells in the presence of FCS, whereas a trend was observed between 2′-FL-treated cells and cells treated with both 2′-FL and UT in AhR KO cells in the absence of FCS (*p* = 0.0589).

CYP1A1 and HMOX-1 mRNA expression was measured in WT (Figure 6a,b) and AhR KO (Figure 7a,b) cells under FCS and serum-free conditions. mRNA expression of both genes was similar across all treatments and cell types under both conditions.

### 2.7. Metabolic Activity and Cytotoxicity Are Not Impacted by UT or 2′-FL Exposure

**Figure 8:**
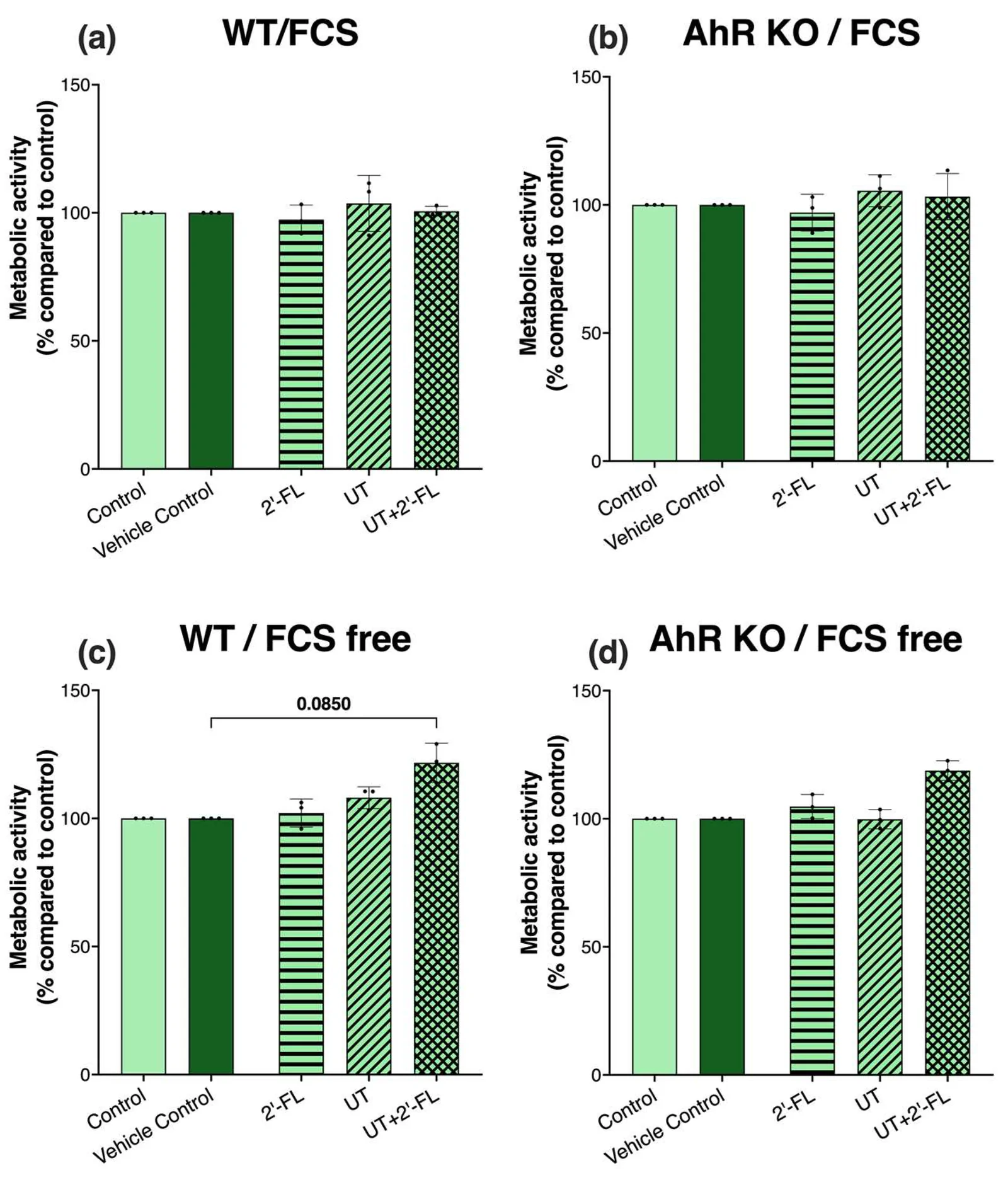
The combination of UTs and 2′-FL does not alter metabolic activity in Caco-2 cells. Metabolic activity is shown for WT (**a**,**b**) and AhR KO (**c**,**d**) Caco-2 cells under different exposures, in the presence (**a**,**c**) and absence (**b**,**d**) of FCS. Data are presented as mean ± SD (*N* = 3, *n* = 2). Within each cell type and FCS condition, treatment-specific effects were assessed using Friedman’s test with Dunn’s post hoc test. Significant overall treatment effects were observed in WT (*p* = 0.0244) and AhR KO (*p* = 0.0392) cells in the absence of FCS; however, post hoc analysis did not identify significant differences between individual treatment groups.

**Figure 9:**
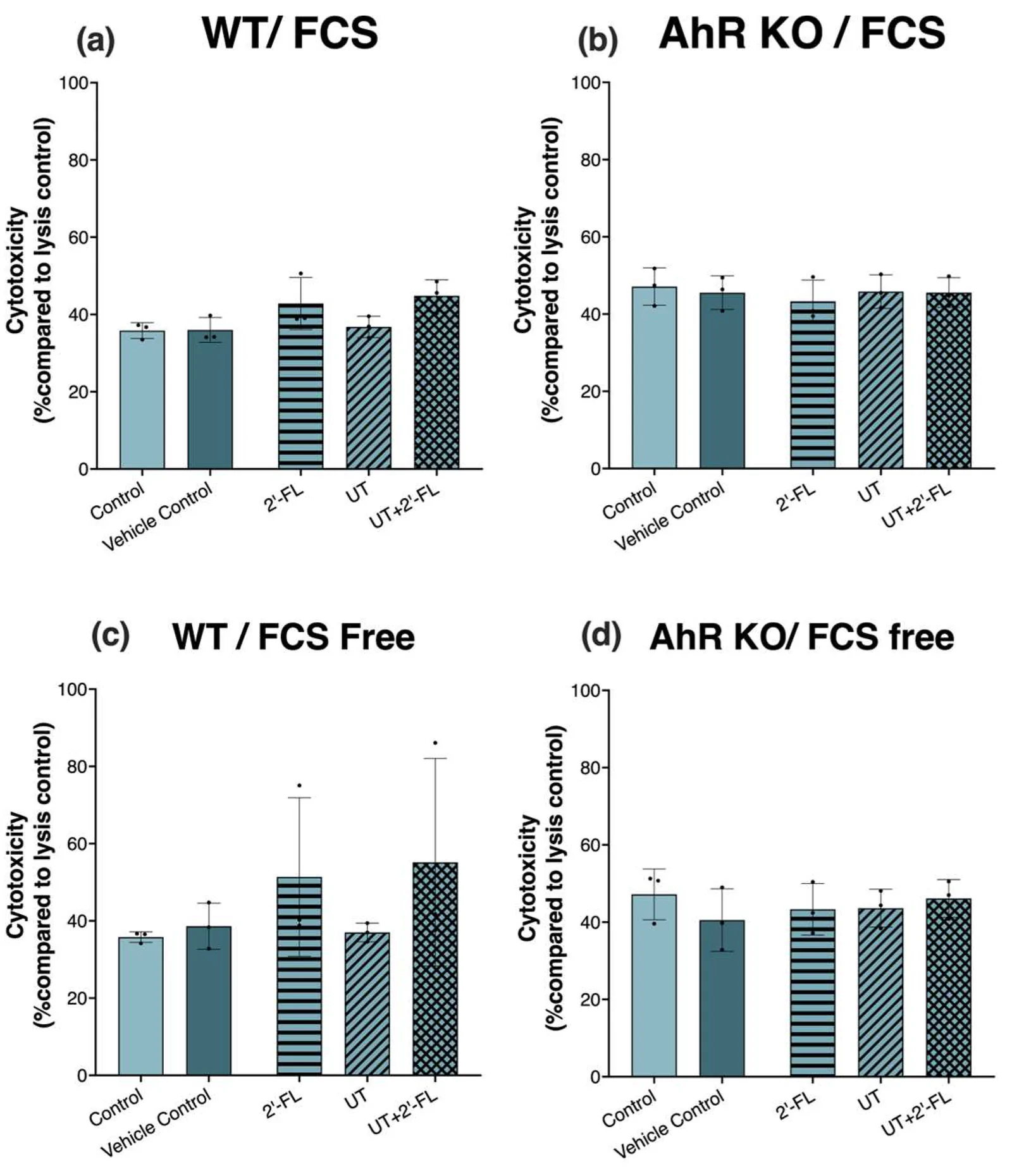
WT and AhR KO Caco-2 cells show no overt cytotoxicity under different exposures. Cytotoxicity is shown for WT (**a**,**c**) and AhR KO (**b**,**d**) cells in the presence (**a**,**b**) and absence (**c**,**d**) of FCS. Data are presented as mean ± SD (*N* = 3, *n* = 2). Within each cell type and FCS condition, treatment-specific effects were assessed using Friedman’s test with Dunn’s post hoc test. A significant overall effect was observed in both cell types in the absence of FCS (*p* = 0.0151), although post hoc analysis did not reveal significant differences between treatment groups.

Metabolic activity (PrestoBlue) and cytotoxicity (LDH release) were assessed in WT and AhR KO cells. PrestoBlue measurements showed similar metabolic activity across all treatments in both WT and AhR KO cells under FCS and serum-free conditions (Figure 8a–d). LDH release was low under all conditions, indicating minimal cytotoxicity (Figure 9a–d).

## 3. Discussion

Our findings support an important role for AhR in intestinal epithelial barrier development and demonstrate that 2′-FL can reduce paracellular permeability under specific experimental conditions. AhR KO cells showed consistently impaired barrier formation during the 21-day differentiation period compared with WT cells, while 2′-FL reduced paracellular permeability in both cell types in the presence of FCS. However, acute exposure to the uremic toxin mixture did not produce a measurable impairment of barrier function. Therefore, the observed effect of 2′-FL should be interpreted as modulation of epithelial permeability rather than rescue of an experimentally induced uremic toxin-mediated barrier defect.

To comprehensively evaluate barrier function, we employed complementary assays targeting different aspects of epithelial integrity: TEER for overall barrier formation, FITC- inulin flux for macromolecular permeability, tight junction gene expression for structural components, canonical AhR target genes as well as metabolic activity and cytotoxicity. This multi-parameter approach was essential given that barrier dysfunction in CKD involves both structural and functional alterations that may not be captured by any single measurement.

The consistent impairment in barrier formation observed in AhR KO cells over the 21-day differentiation period supports an important role for AhR in epithelial barrier development. These results support previous reports linking AhR to epithelial differentiation and mucosal homeostasis [18] while further demonstrating that AhR’s role remains critical throughout the entire course of differentiation [19].

Beyond supporting baseline barrier formation, AhR may also contribute to adaptive responses to environmental stress. In WT cells, the transient decreases in TEER under FCS conditions may reflect dynamic remodeling of tight junctions in response to serum-derived signals. The relative stability of TEER under FCS-free conditions may reflect differences in the cellular environment between serum-containing and serum-free exposure conditions.

In contrast, AhR KO cells exhibited less predictable barrier responses. Although TEER generally increased in these cells, the magnitude and direction of change varied across treatments, suggesting that compensatory pathways, potentially including Nrf2 [20] or HIF-1α [21], may partially contribute to barrier maintenance in the absence of AhR. These findings highlight that AhR does more than support structural components of the barrier. The variability observed in AhR KO cells suggests that AhR contributes to coordinated epithelial barrier responses during environmental stress.

While TEER measurements revealed the complexity of AhR’s regulatory role, paracellular permeability assessments using FITC-inulin flux provided complementary insights into barrier selectivity and the therapeutic potential of 2′-FL. Unlike TEER, which measures electrical resistance, the Papp assay directly quantifies macromolecular passage through the paracellular space, offering a functional, more clinically relevant measure of barrier function in CKD. As these assays assess distinct aspects of epithelial barrier function, differences between TEER and FITC-inulin permeability do not necessarily represent conflicting findings. This distinction is particularly important in CKD, where increased intestinal permeability to bacterial products drives systemic inflammation [10].

Notably, 2′-FL reduced paracellular permeability in both WT and AhR KO cells when FCS was present. This finding demonstrates that the observed permeability response can occur in the characterized AhR-deficient clone under the conditions tested. However, because direct protein-level confirmation of AhR loss was not performed and only a single knockout clone was evaluated, these findings do not establish a definitive AhR-independent mechanism. Further studies using direct protein-level validation, independent AhR knockout clones, and functional validations using an AhR agonist or rescue approaches will be required to determine the contribution of AhR signaling to the response.

The dependence of the 2′-FL-associated permeability response on the presence of FCS also warrants consideration. Although 2′-FL reduced paracellular permeability in both WT and AhR KO cells in the presence of FCS, a comparable effect was not observed under serum-free conditions. FCS provides growth factors and other components that may influence epithelial cell responses; in addition, serum proteins can bind uremic solutes and thereby alter their free concentrations, cellular availability, and biological activity [22]. Because several of the UTs included in the present mixture are substantially protein-bound, differences in serum conditions may therefore influence the effective exposure of the epithelial monolayer to individual toxins [22]. Consequently, the FCS dependence of the observed 2′-FL response cannot be attributed solely to intrinsic epithelial barrier biology, and the contribution of serum-mediated changes in toxin availability remains unresolved. This represents an important limitation of the present experimental design.

However, UTs alone did not significantly alter either TEER or paracellular permeability, which is unexpected given clinical evidence of increased intestinal permeability in CKD [23,24]. The absence of measurable barrier disruption following acute UT exposure may reflect several aspects of the experimental model. First, the 24 h exposure period may have been insufficient to reproduce the progressive barrier dysfunction associated with the chronic nature of CKD. Second, the selected UT concentrations, although based on reported circulating concentrations [25], may require additional cofactors or inflammatory and microbial-derived mediators to produce more pronounced effects on intestinal barrier function [25,26]. The simplified in vitro system may also lack cellular and environmental components that contribute to toxin-induced barrier dysfunction in vivo. Thus, the absence of measurable barrier impairment following acute exposure to the selected UT mixture does not exclude a role for these compounds in CKD-associated intestinal dysfunction, but rather indicates that the exposure conditions used here were insufficient to induce detectable barrier disruption in this model.

The functional reduction in paracellular permeability was not accompanied by detectable changes in the transcriptional expression of ZO-1, occludin, or claudin-1. This finding is notable given clinical evidence of altered intestinal tight-junction protein expression in patients with CKD, including reduced occludin and claudin-1 expression [27]. The absence of corresponding changes in tight-junction gene expression suggests that the observed effect of 2′-FL may not be mediated primarily through transcriptional regulation under the conditions tested. Instead, tight-junction function can also be regulated through changes in protein localization, assembly, or stabilization [28]. Such mechanisms were not investigated in the present study and therefore remain speculative. The relatively short 24 h exposure period may also have been insufficient to capture delayed transcriptional responses associated with chronic CKD-related barrier dysfunction. More broadly, post-translational regulation of tight-junction proteins, including phosphorylation and other modifications that affect protein localization, stability, and function, can alter epithelial barrier properties independently of changes in gene expression [28].

Similarly, assessment of the canonical AhR target genes CYP1A1 and HMOX-1 did not provide clear evidence of transcriptional activation under the conditions tested. CYP1A1 remained largely unchanged across treatments, while HMOX-1 exhibited some variation, but without statistically significant differences. These findings suggest that, in this acute in vitro model, UTs may act as relatively weak AhR agonists, or that additional cofactors, stress signals, or longer exposures may be required for robust transcriptional responses [29,30]. Importantly, both genes are subject to regulation by multiple pathways, including PXR, CAR, Nrf2, and MAPK, which may modulate or mask AhR-dependent effects [31,32,33,34], particularly under metabolically challenging, FCS-free conditions. Together, these observations indicate that, although AhR plays a central role in barrier regulation, the acute transcriptional responses of canonical targets may not fully capture its functional contributions in epithelial cells.

To confirm that observed changes in barrier function were not secondary to cell damage or altered metabolism, we measured cellular cytotoxicity via LDH release and metabolic activity via PrestoBlue. Both parameters remained unchanged across all conditions, indicating that the functional and transcriptional changes were independent of cell death or metabolic impairment. Importantly, even in the absence of FCS, a condition that removes key growth factors and nutrients, both WT and AhR KO cells showed no signs of cytotoxicity or metabolic disruption. These findings suggest that the observed changes in barrier function were not attributable to overt cytotoxicity or metabolic impairment. Nevertheless, the lack of corresponding improvements in barrier function despite maintained metabolic activity suggests that energetic support alone is insufficient to restore epithelial integrity under uremic stress. This disconnect highlights the complexity of barrier regulation and underscores the potential need for therapeutic strategies that target multiple aspects of epithelial function simultaneously.

Several important limitations must be considered when interpreting these results. First, our simplified 2D monolayer system cannot fully recapitulate the complexity of the intestinal environment, which includes dynamic immune-epithelial interactions, microbial communities, and mechanical forces. These factors are particularly relevant in CKD, where barrier dysfunction results from the interplay between uremic toxicity and chronic systemic inflammation. Second, the relatively small number of independent biological replicates (*N* = 3) limits the statistical power and precision of the study, particularly for detecting subtle effects. While we observed changes in paracellular permeability under selected conditions, these findings require validation in larger and more robust experimental designs. In addition, although the selected AhR knockout clone was characterized using Sanger sequencing together with ICE and TIDE analyses, direct confirmation of AhR protein loss was not performed. Therefore, complete loss of AhR protein and the possibility of clone-specific effects cannot be definitively excluded.

Furthermore, experiments were conducted in a single epithelial cell line, limiting extrapolation to more complex intestinal systems. Finally, our acute 24 h exposure paradigm may not adequately reflect the chronic nature of CKD pathophysiology, potentially missing delayed or cumulative effects that develop over extended timeframes.

Despite these limitations, our findings support an important role for AhR in intestinal barrier development and demonstrate that 2′-FL can reduce paracellular permeability under selected experimental conditions. Because acute UT exposure did not induce measurable barrier dysfunction in the present model, the ability of 2′-FL to restore a toxin-induced barrier defect could not be directly evaluated. To further investigate the potential of 2′-FL to modulate intestinal barrier function. Dose–response studies with graded AhR activation and chronic exposure paradigms will better reflect clinical conditions, while the use of patient-derived uremic plasma or more comprehensive toxin panels may provide clinically relevant insights into barrier dysfunction mechanisms.

Advanced experimental models, including intestinal organoids, gut-on-chip platforms, and multi-cellular co-cultures incorporating immune cells and microbiota, will be essential for dissecting the complex interactions between epithelial cells, inflammatory mediators, and microbial metabolites. Together with these models, assessment of tight junction protein expression and localization, alongside pharmacological modulation of AhR signaling, could provide further mechanistic insight into the functional responses observed here. These approaches will ultimately determine whether 2′-FL or structurally related compounds can meaningfully support gut barrier function in patients with CKD and whether targeted AhR modulation represents a viable therapeutic strategy for preventing CKD-associated systemic inflammation and disease progression.

## 4. Materials and Methods

### 4.1. Caco-2 Cell Culture

The human colorectal adenocarcinoma cell line Caco-2 (ATCC HTB-37; Manassas, VA, USA) was cultured in Dulbecco’s Modified Eagle Medium (DMEM; Thermo Fisher Scientific, Waltham, MA, USA; Cat# 42430025) supplemented with 0% FCS (FCS; Bio West, Bradenton, FL, USA; Cat# S1810-500) and 1:100 penicillin/streptomycin (P/S; Thermo Fisher Scientific; Cat# 15140163). Cells were maintained in T75 flasks (Greiner Bio-One, Frickenhausen, Germany; Cat# 658175) at 37 °C in a humidified incubator (Panasonic MCO-20AIC; Marshall Scientific, Hampton, NH, USA) with 5% CO_2_. Medium was refreshed every 2 days, and once Caco-2 cells reached 80–90% confluency, they were harvested using 0.05% trypsin-EDTA (Thermo Fisher Scientific; Cat# 25300-062) for 5 min. WT and AhR KO cells were maintained under identical culture conditions throughout all experiments.

### 4.2. 2D Intestinal Model

Caco-2 cells were seeded onto Thincert cell culture inserts (Greiner Bio-One; Cat# 665641) at a density of 2.5 × 10^4^ cells per insert. Cells were allowed to form a confluent monolayer and differentiate for 21 days in complete culture medium containing 10% FCS. Following the 21-day differentiation period, cells were washed with PBS and were exposed for 24 h to the UT mixture in the basolateral compartment under either complete (10% FCS) or serum-free conditions, in concentrations depicted in Table 1. The composition and concentrations of UTs were selected [25] based on reported circulating concentrations observed in patients with advanced CKD, as documented in the EUTOX database (https://database.uremic-toxins.org/home.php Accessed 1 September 2026), which reports uremic metabolite serum levels in patients with CKD. 2′-fucosyllactose (2′-FL; Jennewein Biotechnologie GmbH, Rheinbreitbach, Germany; Cat# 16103019) was added to the apical compartment at a final concentration of 0.5% for 24 h. This concentration was selected based on previous in vitro studies investigating the effects of 2′-FL on intestinal epithelial barrier function [35]. Vehicle controls contained 0.2% dimethyl sulfoxide (DMSO) and 0.1% sodium hydroxide (NaOH), corresponding to the solvent of UTs. After exposure, transepithelial electrical resistance (TEER), fluorescein isothiocyanate (FITC)-inulin flux, cytotoxicity, metabolic activity, and mRNA expression were assessed.

### 4.3. Transepithelial Electrical Resistance (TEER) Measurement

TEER was measured using a Millicell ERS-2 epithelial Volt-Ohm meter (Merck KGaA, Saint Louis, MO, USA; Cat# MERS00002) before (T0) and after (T1) exposure. To minimize variability arising from incomplete monolayer formation, only inserts with baseline TEER values between 400 and 900 Ω were included. TEER values were normalized to insert surface area, and changes (ΔTEER) were calculated using the following equation:

Δ TEER = TEER_T1_ − TEER_T0_

### 4.4. FITC-Inulin Flux Assay

FITC-inulin (Merck KGaA; Cat# F3272-1G) was prepared as a 10 mg/mL stock solution in phosphate-buffered saline (PBS; Merck KGaA; Cat# D8537) and diluted to 6 μg/mL in Hank’s Balanced Salt Solution (HBSS; Thermo Fisher Scientific; Cat# 14025-100). The FITC-inulin solution was added to the apical compartment, while HBSS was added to the basolateral compartment. After 4 h, the fluorescence of basolateral samples was measured (excitation: 492 nm, emission: 518 nm) using a microplate reader (Fluoroskan Ascent FL; Thermo Fisher Scientific). Apparent permeability (Papp) was calculated using the following equation:

Papp (cm/s) = dQ/dt × 1/(A × C_0_)

where dQ/dt represents the flux rate of FITC-inulin, A is the insert surface area, and C0 is the initial FITC-inulin concentration in the apical compartment [36].

### 4.5. Metabolic Activity Assay

Metabolic activity was assessed using a PrestoBlue assay (Thermo Fisher Scientific; Cat# A13261). Apical medium was replaced with fresh culture medium containing 10% PrestoBlue, while basolateral medium was replaced with fresh culture medium alone. After 1 h at 37 °C, fluorescence of apical samples was measured (excitation: 530 nm, emission: 590 nm) using a microplate reader (Fluoroskan Ascent FL; Thermo Fisher Scientific). Relative metabolic activity was calculated using the following equation:

Relative metabolic activity (%) = 100 × (Experimental metabolic activity)/(Control metabolic activity)

### 4.6. Cytotoxicity Assay

Cytotoxicity was assessed using an LDH assay (Promega; Cat# G1780, Madison, WI, USA). Supernatants were incubated with LDH substrate for 30 min, followed by the addition of stop solution. Absorbance was measured at 492 nm using a microplate reader (Promega, GloMax Discover). Relative cytotoxicity was calculated using the following equation:

Relative cytotoxicity (%) = 100 × (Experimental LDH release/Maximum LDH release)

### 4.7. mRNA Expression Analysis

Following exposure, cell lysates were collected in RLT buffer (Qiagen; Cat# 79216, Hilden, Germany) containing 143 mM β-mercaptoethanol (Thermo Fisher Scientific; Cat# 31350-010) and stored at −80 °C. RNA was extracted using the RNeasy kit (Qiagen; Cat# 74106) and converted to cDNA using the iScript cDNA synthesis kit (Bio-Rad; Cat# 1708891, Hercules, CA, USA). Quantitative PCR (qPCR) was performed with SsoAdvanced Universal SYBR Green Supermix (Bio-Rad; Cat# 1725272) and human gene-specific primers (Table 2). Gene expression was normalized to the housekeeping gene β-actin by calculating ΔCt (Ct_target_ − Ct_β-actin_). Relative expression was subsequently calculated as:

Relative gene expression = 2^^(−Δct)^

### 4.8. CRISPR/Cas9 Gene Editing

To investigate the role of AhR, we genetically engineered Caco-2 cells and generated a Caco-2 line containing an AhR KO using the CRISPR/Cas9 system. AhR CRISPR RNA (crRNA) targeting exon 2 of the AhR gene (5′-TCACCTACGCCAGTCGCAAG-3′) and fluorescently labeled trans-activating CRISPR RNA (tracrRNA) were purchased from IDT (Integrated DNA Technologies, Leuven, Belgium). Primers for DNA amplification, shown in Table 3, were designed using NCBI (Fwd: 5′-CGGGAAGCACCCTGGATTTA-3′, Rvs: 5′-GCCTCCAAACCTCGATTCCA-3′) and ordered from Thermo Fisher Scientific.

### 4.9. DNA Sequencing

Genomic DNA was extracted using the QIAamp DNA Mini Kit (Qiagen, 51304), and DNA concentration was quantified using a NanoDrop spectrophotometer (Thermo Fisher Scientific, NanoDrop One). PCR amplification was performed with Q5 Hot Start High-Fidelity Master Mix (New England Biolabs, M0494S, Ipswich, MA, USA), 200 ng of genomic DNA, nuclease-free water (Qiagen), and AhR-specific primers (Fwd: 5’-CGGGAAGCACCCTGGATTTA-3’, Rvs: 5’-GCCTCCAAACCTCGATTCCA-3’) (Thermo Fisher Scientific). PCR products were purified using the QIAquick PCR Purification Kit (Qiagen, 28104) and sequenced via Sanger sequencing (Macrogen, Amsterdam, The Netherlands) to confirm amplification specificity.

### 4.10. Nucleofection

Following validation of primer specificity, nucleofection was performed. Caco-2 cells were pretreated with the ROCK inhibitor mix RevitaCell (Thermo Fisher Scientific, A2644501) for 1 h at 1:100 in culture medium. Duplex RNA (sgRNA) was prepared by annealing crRNA with fluorescently labeled trans-activating CRISPR RNA (tracrRNA, Integrated DNA Technologies, ATTO550) at a 1:1 molar and volume ratio in Nuclease-Free Duplex Buffer (Integrated DNA Technologies, 11-05-01-12), and heating at 95 °C for 5 min. The ribonucleoprotein (RNP) complex was assembled by incubating duplex RNA with recombinant Cas9 plus protein from Streptococcus pyogenes (SpCas9, Merck KGaA, CAS9PL) for 20 min, which was subsequently supplemented with Alt-R Cas9 electroporation enhancer (Integrated DNA Technologies, 10007805).

Caco-2 WT cells were collected and resuspended in AMAXA buffer (Lonza, Cell Line Nucleofector Kit T, #VCA-1002) before nucleofection using the Nucleofector device (Lonza, Basel, Switzerland). Following nucleofection, cells were cultured overnight in RevitaCell-supplemented medium, which was replaced with fresh medium the following day. A negative control (cells nucleofected without RNP) was included to account for cellular stress and death induced by the procedure.

### 4.11. Clone Selection

Seven days post-nucleofection, single-cell sorting was performed using a FACSAria Fusion flow cytometer (BD Biosciences, Franklin Lakes, NJ, USA) at the Flow Cytometry Facility, Faculty of Science, Utrecht University (Utrecht, The Netherlands). Prior to sorting, cells were incubated with the ROCK inhibitor Y-27632 (Selleckchem, S1049, Houston, TX, USA) for 1 h and filtered through a 70 μm cell strainer (VWR, 734-0003). Single cells were sorted into 96-well plates (Greiner Bio-One, 655191) and centrifuged at 400× *g* for 1 min. Clonal expansion was monitored, and once colonies reached 25% confluence, they were transferred from 96-well plates to 12-well plates (Corning, 3512, Corning, NY, USA) and subsequently to T25 flasks (Greiner Bio-One, 690175). At ~80% confluence, genomic DNA was extracted and analyzed via Sanger sequencing. Mutation analysis was performed using the ICE (Synthego, Menlo Park, CA, USA) and TIDE web tools [37] to confirm successful AhR KO or negative control clones. Verified Caco-2 AhR KO and WT clones were subsequently utilized in the 2D intestinal model.

### 4.12. Statistical Analysis

Statistical analyses were performed using GraphPad Prism 10.2.2 (GraphPad Software, Boston, MA, USA). Unless otherwise stated, experiments were performed using three independent biological replicates (*N* = 3), with two technical replicates per condition (*n* = 2). Technical replicates were averaged within each biological replicate before statistical analysis, such that the biological replicate remained the experimental unit. Outliers were identified using the robust regression and outlier removal (ROUT) method (Q = 1%).

Treatment conditions were matched within each independent biological experiment, with each biological replicate contributing data to all treatment conditions. As the data comprised matched repeated measures and the limited number of biological replicates (*N* = 3) did not permit reliable assessment of the assumptions required for parametric analysis, the Friedman test was used as the non-parametric alternative to repeated-measures ANOVA. Analyses were performed separately for each cell line and FCS condition for TEER, FITC-inulin permeability, tight junction gene expression, AhR canonical gene expression, PrestoBlue metabolic activity, and LDH release. Where an overall treatment effect was identified, Dunn’s multiple-comparisons test was used for post hoc pairwise comparisons. The prespecified comparisons were as follows: Control versus Vehicle Control, Control versus 2′-FL, Vehicle Control versus UT, Vehicle Control versus UT + 2′-FL, 2′-FL versus UT + 2′-FL, and UT versus UT + 2′-FL. Dunn’s procedure accounted for multiple pairwise comparisons within each analysis; no additional correction was applied across the separate endpoint-level analyses.

TEER development over time was analyzed using a mixed-effects model with restricted maximum likelihood (REML), followed by Tukey’s multiple-comparisons test, to account for repeated measurements from the same biological replicates over the differentiation period and to assess differences between cell lines over time. Data are presented as mean ± standard deviation (SD). A *p*-value ≤ 0.05 was considered statistically significant.

## Figures and Tables

**Table 1:** Composition and concentrations of UTs.

Compound	UT (μΜ)
Indoxyl Sulphate	100
Indole-3-acetic Acid	10
Indoxyl-β-δ-glucuronide	10
Kynurenic Acid	1
L-Kynurenine	3
Hippuric Acid	600
3-Carboxy-4-Methyl-5-Propyl-Furanpropionic Acid	100
P-Cresyl Glucuronide	40
P-Cresyl Sulfate	125

**Table 2:** Genes of interest and sequences.

Gene of Interest	Forward (5′ → 3′)	Reverse (5′ → 3′)
β-actin	CTGGAACGGTGAAGGTGACA	AAGGGACTTCCTGTAACAATGCA
ZO-1	CAAGATAGTTTGGCAGCAA	ATCAGGGACATTCAATAGC
Occludin	TGTGGATCCCCAGGAGGCC	AGGCACGTCCTGTGTGCCT
Claudin-1	GGGCTGCAGCTGTTGGGCT	GGGTTGCTTGCAATGTGCT
AhR	CAAATCCTTCCAAGCGGCA	CGCTGAGCCTAAGAACTGA
CYP1A1	ATGAACCCCAGGGTACAGAGAA	AAGCCTTTCAAACTTGTGTCTCTT
HMOX-1	ATGGCCTCCCTGTACCACATC	TGTTGCGCTCAATCTCCTCCT

**Table 3:** CRISPR/Cas9 target sequences.

Component	Sequence (5′ -> 3′)
cRNA	TCACCTACGCCAGTCGCAAG
Forward Primer	CGGGAAGCACCCTGGATTTA
Reverse Primer	GCCTCCAAACCTCGATTCCA

## Data Availability

The original contributions presented in this study are included in the article/Supplementary Material. Further inquiries can be directed to the corresponding author.

## Supplementary Materials

The following supporting information can be downloaded at: https://www.mdpi.com/article/10.3390/toxins18100451/s1. Figure S1: Generation and characterization of AhR knock-out (KO) and wild-type (WT) control Caco-2 cell lines using CRISPR-Cas9. (a) Schematic overview of the CRISPR-Cas9 workflow used to generate AhR WT and KO clones in human colonic Caco-2 cells. Created in BioRender. Masereeuw, R. (2026) https://BioRender.com/tzgu57r, accessed on 12 October 2026. (b) Sanger sequencing chromatograms showing the WT control. (c) Sanger sequencing chromatograms showing disruption of the AhR target sequence in the selected KO clone. (d) Predicted protein structures derived from the WT control. (e) Predicted protein structures derived from the edited sequences support the predicted loss of functional AhR protein in the KO clone. WT, wild-type; KO, knock-out; AhR, aryl hydrocarbon receptor.

## Abbreviations

The following abbreviations are used in this manuscript:

2′-FL	2′-Fucosyllactose
AhR	Aryl hydrocarbon receptor
ATCC	American Type Culture Collection
CAR	Constitutive androstane receptor
Caco-2	Human colorectal adenocarcinoma cell line
CKD	Chronic kidney disease
CRISPR	Clustered regularly interspaced short palindromic repeats
Cas9	CRISPR-associated protein 9
cDNA	Complementary DNA
CO_2_	Carbon dioxide
Ct	Cycle threshold
CYP1A1	Cytochrome P450 family 1 subfamily A member 1
DMEM	Dulbecco’s Modified Eagle’s Medium
DMSO	Dimethyl sulfoxide
DNA	Deoxyribonucleic acid
EUTOX	European Uremic Toxin Work Group
FACS	Fluorescence-activated cell sorting
FCS	Fetal calf serum
FITC	Fluorescein isothiocyanate
gRNA	Guide RNA
HBSS	Hank’s Balanced Salt Solution
HIF-1α	Hypoxia-inducible factor 1-alpha
HMOX-1	Heme oxygenase 1
ICE	Inference of CRISPR Edits
I3AA	Indole-3-acetic acid
IS	Indoxyl sulfate
KO	Knockout
LDH	Lactate dehydrogenase
MAPK	Mitogen-activated protein kinase
mRNA	Messenger RNA
NaOH	Sodium hydroxide
Nrf2	Nuclear factor erythroid 2-related factor 2
PBS	Phosphate-buffered saline
Papp	Apparent permeability coefficient
P/S	Penicillin/streptomycin
PXR	Pregnane X receptor
qPCR	Quantitative polymerase chain reaction
REML	Restricted maximum likelihood
RNP	Ribonucleoprotein
ROCK	Rho-associated coiled-coil-containing protein kinase
ROUT	Robust regression and outlier removal
SD	Standard deviation
SEM	Standard error of the mean
sgRNA	Single-guide RNA
SpCas9	Streptococcus pyogenes Cas9
TEER	Transepithelial electrical resistance
TIDE	Tracking of Indels by DEcomposition
TJ	Tight junction
UT	Uremic toxin
WT	Wild type
ZO-1	Zonula occludens-1
