# Supplementary material for: Aryl Hydrocarbon Receptor Deficiency Impairs Intestinal Barrier Development, While 2′-Fucosyllactose Reduces Paracellular Permeability in Caco-2 Cells
Source: Toxins (Basel). 2026 Oct 15;18(10):451. doi: 10.3390/toxins18100451 (PMC13709715; doi:10.3390/toxins18100451)
Supplement: Supplementary file 1 [file toxins-18-00451-s001.zip › toxins-4566086-supplementary.pdf]

Supplementary Materials: Aryl Hydrocarbon Receptor Deficiency Impairs Intestinal Barrier Development, While 2′-Fucosyllactose Reduces Paracellular Permeability in Caco-2 Cells

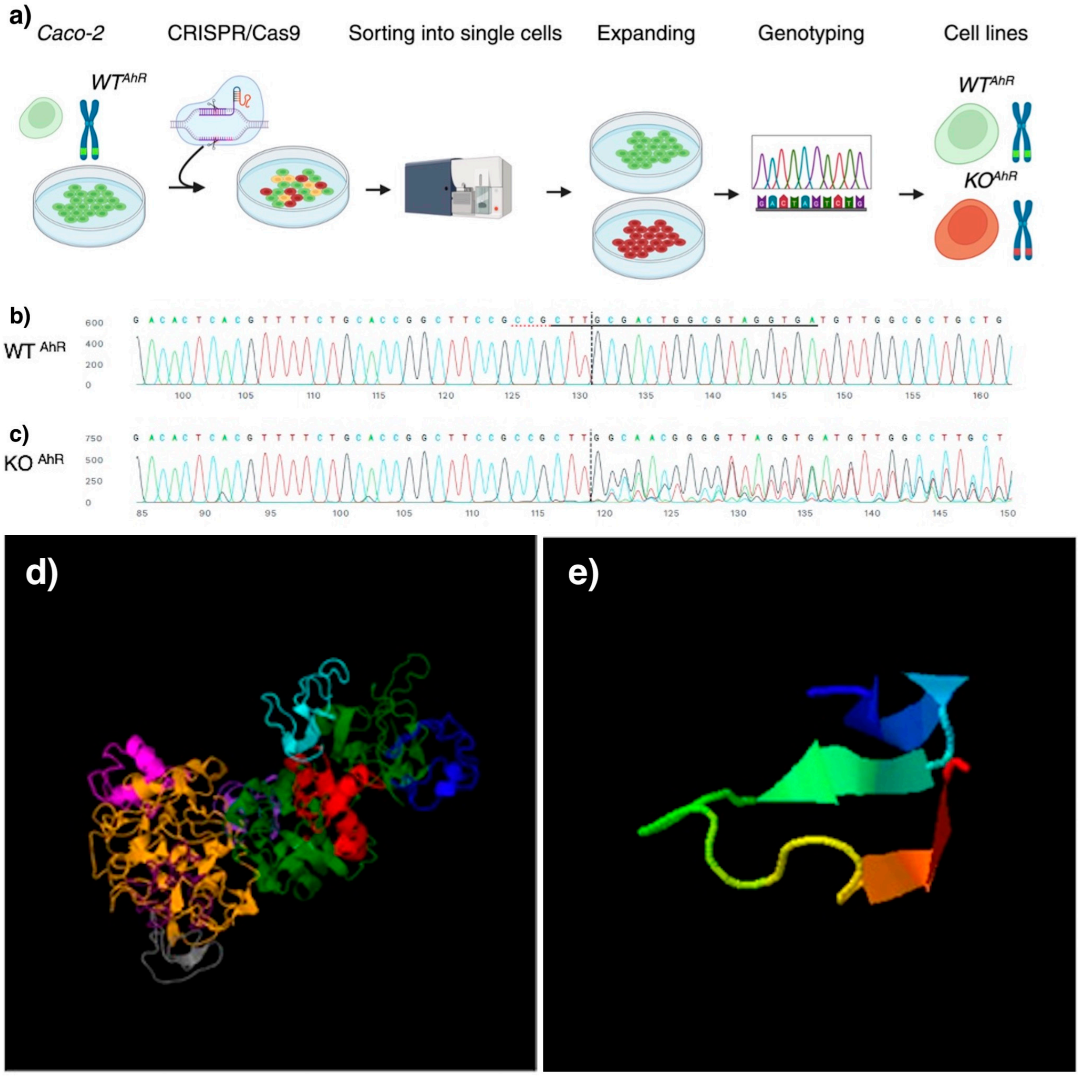

Figure S1. Generation and characterization of AhR knock-out (KO) and wild-type (WT) control Caco-2 cell lines using CRISPR-Cas9. (a) Schematic overview of the CRISPR-Cas9 workflow used to generate AhR WT and KO clones in human colonic Caco-2 cells. Created in BioRender. Masereeuw, R. (2026) https://BioRender.com/tzgu57r (b) Sanger sequencing chromatograms showing the WT control. (c) Sanger sequencing chromatograms showing disruption of the AhR target sequence in the selected KO clone. d) Predicted protein structures derived from the WT control. (e) Predicted protein structures derived from the edited sequences

support the predicted loss of functional AhR protein in the KO clone. WT, wild-type; KO, knock-out; AhR, aryl hydrocarbon receptor.
